# Characterization of the Mitochondrial Genome of *Hippophae rhamnoides* subsp. *sinensis* Rousi Based on High-Throughput Sequencing and Elucidation of Its Evolutionary Mechanisms

**DOI:** 10.3390/plants14162547

**Published:** 2025-08-15

**Authors:** Mengjiao Lin, Na Hu, Jing Sun, Wu Zhou

**Affiliations:** 1 College of Eco-Environmental Engineering, Qinghai University, Xining 810016, China; lmj250702@qhu.edu.cn; 2 Qinghai Provincial Key Laboratory of Qinghai-Tibet Plateau Biological Resources, Northwest Institute of Plateau Biology, Chinese Academy of Sciences, Xining 810008, China; huna@nwipb.cas.cn (N.H.); sunj@nwipb.cas.cn (J.S.)

**Keywords:** *Hippophae rhamnoides* subsp. *sinensis*, mitochondrial genome, repeat sequence, phylogenetic analysis

## Abstract

*Hippophae rhamnoides* ssp. *sinensis* Rousi a species of significant ecological and economic value that is native to the Qinghai–Tibet Plateau and arid/semi-arid regions. Investigating the mitochondrial genome can elucidate stress adaptation mechanisms, population genetic structure, and hybrid evolutionary history, offering molecular insights for ecological restoration and species conservation. However, the genetic information and evolutionary mechanisms of its mitochondrial genome remain poorly understood. This study aimed to assemble the complete mitochondrial genome of *H. rhamnoides* L. ssp. *sinensis* using Illumina sequencing, uncovering its structural features, evolutionary pressures, and environmental adaptability and addressing the research gap regarding mitochondrial genomes within the *Hippophae* genus. The study assembled a 454,444 bp circular mitochondrial genome of *H. rhamnoides* ssp. *sinensis*, with a GC content of 44.86%. A total of 73 genes and 3 pseudogenes were annotated, with the notable absence of the *rps2* gene, which is present in related species. The genome exhibits significant codon usage bias, particularly with high-frequency use of the alanine codon GCU and the isoleucine codon AUU. Additionally, 449 repetitive sequences, potentially driving genome recombination, were identified. Our evolutionary pressure analysis revealed that most genes are under purifying selection, while genes such as *atp4* and *nad4* exhibit positive selection. A nucleotide diversity analysis revealed that the *sdh4* gene exhibits the highest variation, whereas *rrn5* is the most conserved. Meanwhile, phylogenetic analysis showed that *H. rhamnoides* ssp. *sinensis* from China is most closely related to *Hippophae tibetana*, with extensive homologous sequences (49.72% of the chloroplast genome) being identified between the chloroplast and mitochondrial genomes, indicating active inter-organellar gene transfer. Furthermore, 539 RNA editing sites, primarily involving hydrophilic-to-hydrophobic amino acid conversions, were predicted, potentially regulating mitochondrial protein function. Our findings establish a foundation for genetic improvement and research on adaptive evolutionary mechanisms in the *Hippophae* genus, offering a novel case study for plant mitochondrial genome evolution theory.

## 1. Introduction

Mitochondria are essential organelles in eukaryotic cells, functioning as primary sites for energy synthesis and conversion and thus supporting various cellular physiological activities. In the early 1960s, M. and S. Nass et al. [1] first identified genetic material within mitochondria. Subsequently, researchers identified all the essential components for replication, transcription, and protein translation in mitochondria, such as DNA polymerase, RNA polymerase, transfer RNA, and ribosomal RNA. These findings collectively demonstrate that mitochondria possess a relatively independent genetic transcription system [2]. In-depth study of the plant mitochondrial genome aids in elucidating the molecular mechanisms of energy metabolism in plant cells, understand plants’ adaptive evolutionary changes, and decipher phylogenetic relationships among species.

The evolutionary dynamics of plant mitochondrial genomes are a widely studied topic. Compared to the conserved plastid genomes of plants and the compact mitochondrial genomes of animals, plant mitochondrial genomes exhibit unique characteristics [3]. The size, gene composition, and structure of these genomes vary significantly across plant groups. Frequent events such as gene loss, gene transfer to the nuclear genome, and structural variations drive the continuous evolution of these genomes [4,5,6]. The size of fully sequenced plant mitochondrial genomes varies 100-fold, ranging from 66 kb in *Viscum scurruloideum* to 11,700 kb in *Larix sibirica* [3,7]. This significant variation is mainly caused by extensive repetitive sequences and frequent recombination events. Moreover, horizontal gene transfer (HGT) is common in plant mitochondrial genomes. For example, the mitochondrial genome of the angiosperm *Amborella trichopoda* contains up to 20 protein-coding genes, acquired through horizontal transfer from terrestrial plants such as mosses and ferns, including complete mitochondrial genome fragments; this highlights the ability of plant mitochondria to capture exogenous genes [8]. Furthermore, mitochondrial gene transfer has been observed between the parasitic plant *Cuscuta* and its host *Plantago*, integrated through gene conversion [9]. HGT events in plant mitochondrial genomes may occur via parasitic relationships, pollen mediation, or organelle fusion [10].

Recent advancements in high-throughput sequencing technologies, including Illumina, Nanopore, and PacBio, have significantly enhanced the assembly and annotation of plant mitochondrial genomes [11]. To date, the mitochondrial genomes of over 500 plant species have been sequenced, providing extensive data for studying their evolutionary mechanisms and functional diversity. An analysis of the *Taraxacum mongolicum* mitochondrial genome revealed that it forms diverse circular conformations via recombination, mediated by five repeat sequences [12]. These repeat sequences may promote genomic structural rearrangements through homologous recombination, influencing the expression and function of genes. As an independent genetic system, the mitochondrial genome exhibits significant differences in its evolutionary patterns compared to the chloroplast genome [13,14]. This distinction is particularly evident in high-altitude or extreme environments, where mitochondrial genome variation plays a critical role. A study on high-altitude hornwort plants demonstrated that hypoxic conditions significantly accelerated the evolutionary rate of mitochondrial genes, driving adaptive changes in energy metabolic pathways [15].

**Figure 1:**
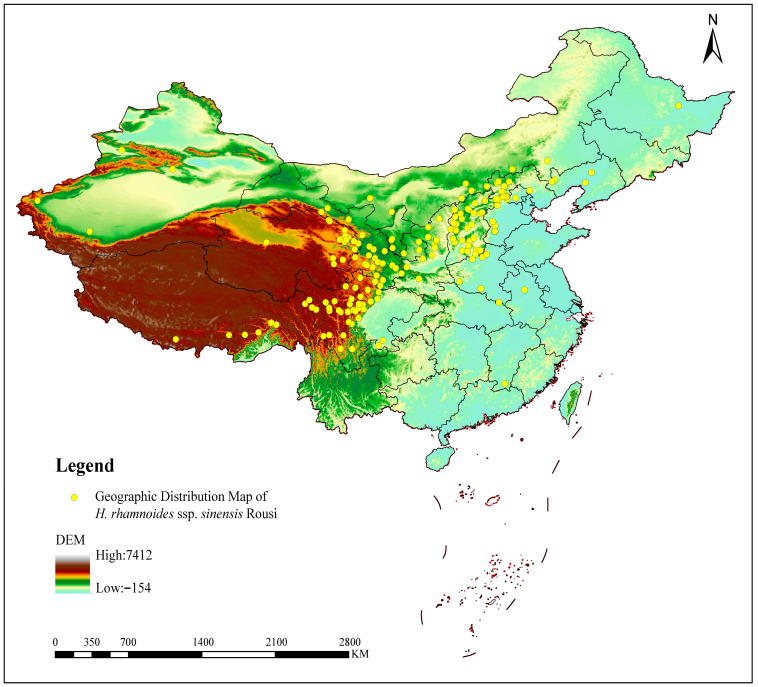
Geographic distribution map of *H. rhamnoides* ssp. *sinensis*.

*Hippophae rhamnoides* ssp. *sinensis*, commonly known as Chinese seabuckthorn, is a subspecies exclusively distributed within China [16,17], with its geographical distribution illustrated in Figure 1. This deciduous shrub or small tree has significant ecological and economic value. It is widely distributed in northwestern, northern, and southwestern China, typically growing on mountain slopes, in valleys, and along riverbanks at elevations ranging from 800 to 4500 m [18]. Ecologically, *H. rhamnoides* ssp. *sinensis* possesses a robust root system and significant nitrogen-fixing abilities, effectively improving soil structure, fertility, and water and nutrient retention [19]. Its dense foliage intercepts rainfall, reducing soil erosion by mitigating the direct impact of raindrops. Furthermore, it exhibits remarkable adaptability to harsh environments, thriving under extreme conditions such as drought, barren soils, cold climates, and high altitudes [20]. This adaptability makes it a pioneer species for vegetation restoration, soil and water conservation, and ecological rehabilitation in China’s arid and semi-arid regions [21]. Economically, the fruit of *H. rhamnoides* ssp. *sinensis* is rich in bioactive compounds, including vitamins, fatty acids, amino acids, and flavonoids, endowing it with high nutritional and medicinal value [22,23]. The fruit can be processed into various products, including juices, wines, jams, and health supplements, highlighting its significant market potential [24]. Extracts like sea buckthorn seed oil and flavonoids are extensively utilized in the pharmaceutical and cosmetic industries [25,26,27].

Despite the significant ecological and economic importance of *H. rhamnoides* ssp. *sinensis*, research has primarily focused on its ecological characteristics, physiological and biochemical mechanisms, chemical composition, and applications, with limited exploration of its genetic information, particularly at the mitochondrial genome level. The mitochondrial genome, as the core genetic material for energy metabolism in plant cells, offers crucial sequence and structural insights into the growth, development, stress adaptation, and evolutionary history of *H. rhamnoides* ssp. *sinensis*. Additionally, the mitochondrial genomes of *Hippophae tibetana* and *Hippophae salicifolia* have been documented [28,29]. The analysis of the complete mitochondrial genome of *H. rhamnoides* ssp. *sinensis* addresses research gaps and provides a robust theoretical foundation for future genetic improvement and cultivar selection of sea buckthorn.

## 2. Results

### 2.1. Sequencing and Genomic Features of the H. rhamnoides ssp. sinensis Mitogenome

**Figure 2:**
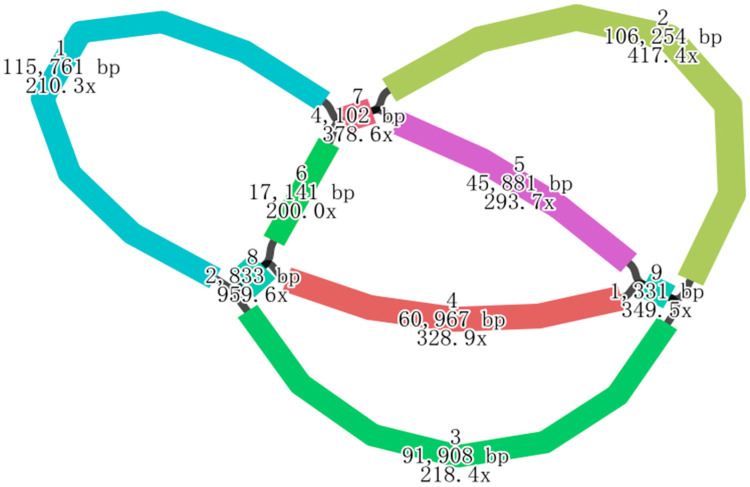
The assembly results for the mitochondrial genome of *H. rhamnoides* ssp. *sinensis*. Numbers 1–9 denote read indices, those ending with “bp” signify read lengths, and those ending with “x” indicate read depths.

**Figure 3:**
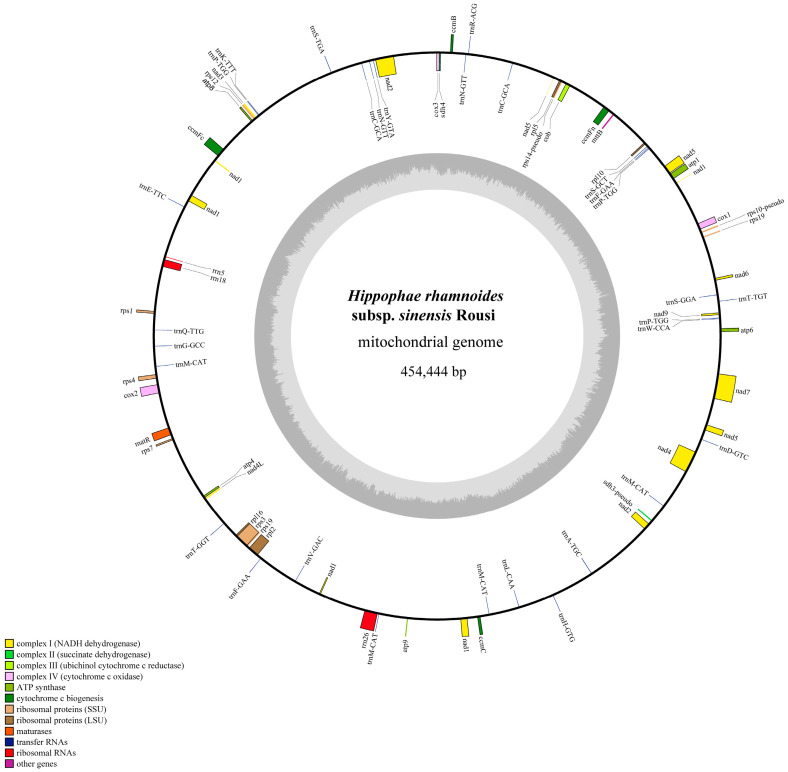
Mitochondrial genome map of *H. rhamnoides* ssp. *sinensis*. Coding genes that are transcribed in the forward direction are displayed on the outer rim of the circle, whereas those that are transcribed in the reverse direction are presented on the inner rim. The innermost gray circle indicates the GC content across the genome.

Whole-genome sequencing of *H. rhamnoides* ssp. *sinensis* generated 20.69 Gb of Illumina data and 16.66 Gb of Nanopore PromethION data, with an average read length of 16,323 bp (Supplementary Material Table S1). The base composition, error rate distribution, and quality score distribution of the Illumina data, as well as the read length distribution of the Nanopore data, are detailed in Supplementary Materials Figures S1–S4. Based on the draft assembly (Figure 2), the mitochondrial genome of *H. rhamnoides* ssp. *sinensis* has been assembled, with a total length of 454,444 bp and a circular structure (Figure 3). The mitochondrial genome exhibits a GC content of 44.86%. A total of 73 genes were annotated, comprising 37 protein-coding genes (PCGs), 30 tRNA genes encoding all standard amino acids, 3 rRNA genes (*rrn18*, *rrn26*, *rrn5*), and 3 pseudogenes (*rps10*, *rps14*, *sdh3*) (Supplementary Materials Table S2). Among the 30 tRNA genes, *trnM-CAT* (4 copies), *trnP-TGG* (3 copies), and *trnN-GTT* (2 copies) show multicopy phenomena, potentially enhancing the mitochondrial translation efficiency. The rRNA genes, including *rrn18*, *rrn26*, and *rrn5*, align with the typical characteristics of angiosperm mitochondrial genomes.

The mitochondrial genome encodes essential components for oxidative phosphorylation (OXPHOS), such as ATP synthase (*atp1*, *atp4*, *atp6*, *atp8*, *atp9*), cytochrome c oxidase (*cox1*, *cox2*, *cox3*), NADH dehydrogenase (*nad1*-*nad9*), and ubiquinol cytochrome c reductase (*cob*) (Supplementary Materials Table S3). Notably, genes such as *ccmFc*, *cox2*, and *rpl2* contain introns, while *nad1* exists in two copies, both harboring introns. Furthermore, *nad4*, *nad5*, and *nad7* are predicted to be RNA editing hotspots, likely undergoing extensive C-to-U conversions. Additionally, *rps10*, *rps14*, and *sdh3* have been rendered nonfunctional pseudogenes, suggesting gene loss events during evolution.

Compared to other *Hippophae* species, the mitochondrial genome of *H. rhamnoides* ssp. *sinensis* exhibits a highly conserved gene order and composition but lacks the *rps2* gene, indicating lineage-specific gene degeneration. Moreover, the presence of multiple tRNA gene copies contrasts sharply with some Rosids, potentially reflecting an adaptive evolutionary response to high-altitude environmental stress. Numerous repeat sequences longer than 1 kb were identified in intergenic regions and are potentially involved in recombination processes. Additionally, some genes utilize non-standard start and stop codons; for example, *nad1* and *nad4L* use ACG as an alternative start codon (potentially edited to AUG post-transcription), while *atp9* and *ccmFc* use CGA as a stop codon (potentially edited to UGA).

### 2.2. Codon Usage Bias Analysis

To investigate codon usage patterns in the mitochondrial genome of the target species, a comprehensive codon preference analysis was conducted. The Relative Synonymous Codon Usage (RSCU) was meticulously calculated, providing precise results (Supplementary Materials Table S4). The analysis revealed that some codons exhibit significant usage frequency preferences. For example, the RSCU value of GCU for alanine (Ala) is 1.5811, indicating a significantly higher usage frequency than other synonymous codons and thus reflecting a strong preference. Similarly, the RSCU value of UUU for phenylalanine (Phe) is 1.1140, indicating a higher usage frequency than for UUC. For isoleucine (Ile), the RSCU value of AUU is 1.3269, reflecting a higher usage frequency. These results demonstrate that codon selection in the mitochondrial genome of this species is non-random and exhibits significant preferences.

**Figure 4:**
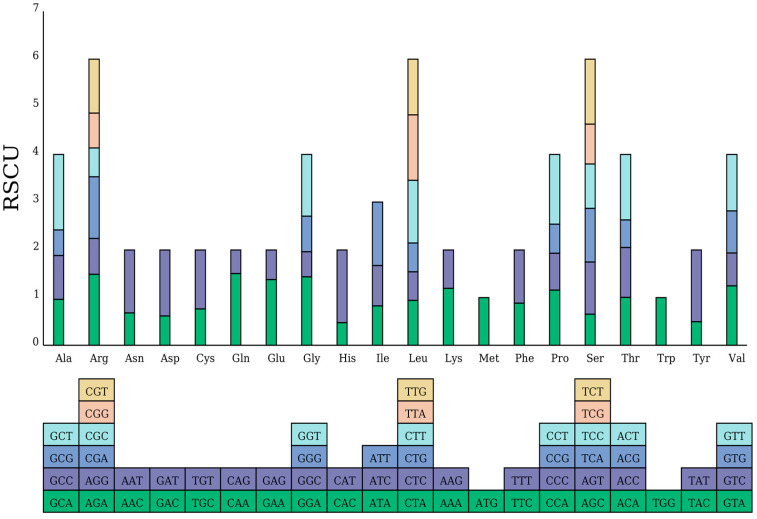
RSCU values for the mitochondrial genome of *H. rhamnoides* ssp. *sinensis*. The x-axis displays different amino acids. The RSCU values represent the observed frequency of each codon relative to its expected frequency under uniform synonymous codon usage.

The codon preference histogram (Figure 4) visually illustrates this preference. The figure reveals significant differences in the total RSCU values of codons for different amino acids. Amino acids such as arginine (Arg), leucine (Leu) and serine (Ser) exhibit higher column heights, reflecting increased codon usage frequency and confirming codon usage bias. This bias is likely influenced by multiple factors, particularly translation efficiency. Codons with higher usage frequencies likely correspond to abundant tRNA species, thereby enhancing translation efficiency and ensuring rapid protein synthesis. Additionally, base composition is a key factor in codon usage bias. A genome’s base composition restricts codon choices, favoring codons that align with its compositional preferences. Natural selection significantly shapes codon usage bias, driving the adoption of patterns that enhance adaptability and survival.

### 2.3. Repeat Sequence Analysis

**Figure 5:**
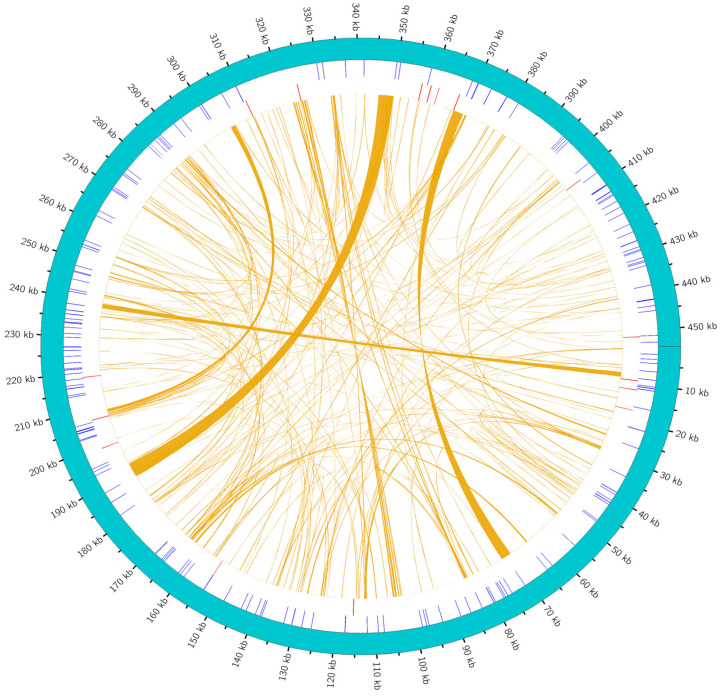
The distribution of repetitive sequences in the mitochondrial genome of *H. rhamnoides* ssp. *sinensis*. The localization of various repeat types along the genome. The outermost circle in sky blue represents the genomic scale. Simple and tandem repeats are shown in blue and red, respectively. Scattered repeats are presented in the innermost circle.

An in-depth analysis of repeat sequences in the mitochondrial genome of *H. rhamnoides* ssp. *sinensis* provided critical insights into its genomic structure, function, and evolutionary mechanisms. This study comprehensively analyzed SSRs, tandem repeats, and interspersed repeats, integrating data to elucidate the complex characteristics of the repeat sequences in the mitochondrial genome of *H. rhamnoides* ssp. *sinensis* (Figure 5). The mitochondrial genome of *H. rhamnoides* ssp. *sinensis* comprises 449 repeat sequences. Among these, 179 were SSRs, 18 were tandem repeats, and 252 were interspersed repeats. Among the 179 SSRs, monomers dominated at 34% (61), while dimers and trimers each constituted 16.7% (30), and tetramers accounted for 29% (52) (Supplementary Materials Figure S5). In monomeric SSRs, the A/T motif dominated, representing 96.7% of the total (Supplementary Materials Figure S6).

Tandem repeats likely play a crucial role in genome organization and functionality. This study identified 18 tandem repeats (Supplementary Materials Table S5), ranging in length from 11 to 39 bp and with copy numbers between 1.9 and 6.3. The similarity of these repeats ranged from 72% to 100%. For example, the 11 bp sequence “TTCTGTTGTTG” had a copy number of 2.3 and 100% similarity. In contrast, the 9 bp sequence “TATTGACGA” had a higher copy number of 6.3 but a lower similarity of 72%. These variations indicate that tandem repeat sequences have undergone distinct selective pressures during evolution, and their diversity may be closely linked to biological processes such as gene expression regulation and genetic variation.

Interspersed repeats are critical in genome evolution, acting as key drivers of genome size variation and structural rearrangements [30]. A total of 252 interspersed repeats were identified, including 117 direct repeats and 88 palindromic repeats within the 0–100 bp range. A sharp decline in repeat numbers occurs with increasing length, with 14 direct and 11 palindromic repeats in the 100–200 bp range, respectively, and fewer in longer ranges (Supplementary Materials Figure S7). Their characteristics, including alignment length, similarity, and start and end positions, exhibit significant diversity. The alignment lengths range from 31 to 4105 bp, with similarities between 74.099% and 100%. Longer alignments with high similarity, such as a 4105 bp alignment with 99.976% similarity, may function in conserved genomic regions, while lower-similarity repeats may have undergone mutations or distinct selective pressures during evolution.

### 2.4. Analysis of Synonymous and Nonsynonymous Replacement Rates

An analysis of different species combinations revealed significant variations in the Ka/Ks ratios of the mitochondrial genes in *H. rhamnoides* ssp. *sinensis*. Comparisons with species such as *Ziziphus jujuba* (NC_029809), *Cannabis sativa* (NC_029855), *Rosa rugosa* (NC_065237), *Hippophae gyantsensis* (NC_086921), *Hippophae tibetana* (PP712939), and *Hippophae salicifolia* (PQ653489) showed average Ka/Ks ratios ranging from 0.21 to 0.72 (Supplementary Materials Table S6). The lowest average Ka/Ks ratio (0.21) was observed in comparison with *Hippophae tibetana*, while the highest (0.72) was found in comparison with NC_086921. These results suggest that the mitochondrial genes of *H. rhamnoides* ssp. *sinensis* have experienced varying degrees of selective pressure across different species.

**Figure 6:**
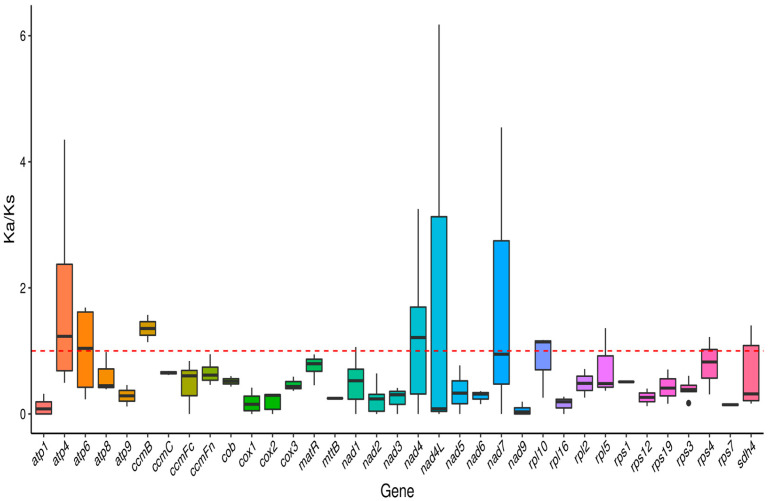
Boxplots showing pairwise Ka/Ks ratios among shared mitochondrial genome genes across 7 species. The red dashed line indicates the neutral expectation of Ka/Ks = 1. Genes with median Ka/Ks values above this line may be under positive selection, while those below are likely evolving under purifying selection.

The Ka/Ks values of different genes show significant variation (Figure 6). For example, the Ka/Ks value of *atp1* ranges from 0 to 0.319122 across species, suggesting that this gene is evolutionarily conserved and under less selective pressure. In contrast, the Ka/Ks value of *atp4* varies significantly, reaching 1.71495 with *Ziziphus jujuba* and 4.34976 with *Cannabis sativa*. This suggests that *atp4* may have undergone strong positive selection, leading to adaptive functional changes. Among the respiratory chain genes, the Ka/Ks values of *cox1*, *cox2*, and *cox3* are predominantly below 1. For example, the Ka/Ks values of *cox1* compared with *Ziziphus jujuba*, *Cannabis sativa*, and *Rosa rugosa* are 0.416316, 0.237583, and 0.0681953, respectively. This indicates that these genes have undergone strong purifying selection, resulting in conserved protein structures and functions that are essential for mitochondrial respiratory chain activity. Additionally, the Ka/Ks values of the *nad* gene family show diversity. Across different species, the Ka/Ks ratio of *nad4* varies significantly, suggesting it may have experienced complex selective pressures and that its function may be closely linked to the adaptive evolution of species.

### 2.5. Nucleotide Diversity

**Figure 7:**
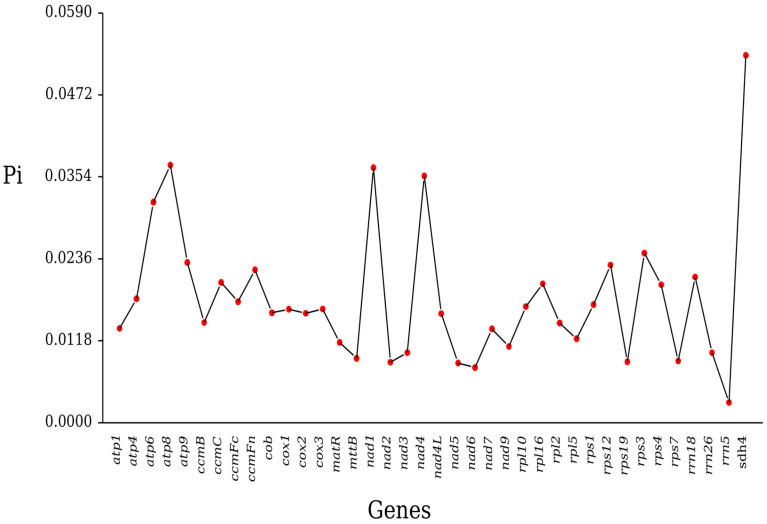
Nucleotide variability (Pi) across genes in the mitochondrial genome of *H. rhamnoides* ssp. *sinensis*.

Our analysis of homologous genes across seven Rosales species revealed that most genes exhibited low nucleotide diversity. The pi values of the genes in the mitochondrial genome of *H. rhamnoides* ssp. *sinensis* exhibited significant variation, ranging from 0.00290 to 0.05291 (Figure 7). This variation indicates that the nucleotide sequence diversity varies among genes. Overall, the pi value distribution reflects non-uniform selection pressures and variation accumulation in mitochondrial genes during evolution.

Among the genes analyzed, *sdh4* exhibited the highest pi value (0.05291). With 61 variable sites and a gene length of 543 bp, *sdh4* demonstrated relatively high nucleotide sequence diversity. This high variability indicates that *sdh4* could serve as a valuable molecular marker in population genetics, with its diversity potentially being linked to adaptive evolution in diverse environments. In contrast, *rrn5* exhibited the lowest pi value (0.00290), with only one variable site and a gene length of 121 bp. This indicates that *rrn5* is highly conserved and under strong selective constraints, suggesting that its sequence stability is essential for mitochondrial function. Within the *atp* gene family, pi values varied among individual genes. The pi value of *atp8* (0.03708) was higher than those of *atp1* (0.01357), *atp4* (0.01785), and *atp9* (0.02307). This suggests that *atp8* is more variable, potentially reflecting unique functional adaptability, while *atp1*, *atp4*, and *atp9* are more conserved, likely maintaining essential energy metabolism. The respiratory chain genes *cox1*, *cox2*, and *cox3* exhibited pi values of 0.01634, 0.01575, and 0.01637, respectively. These low and similar values indicate high conservation of these genes during evolution. The encoded proteins are essential for mitochondrial respiratory chain stability, with even minor sequence variations potentially affecting function and imposing stringent selective constraints.

### 2.6. Phylogenetic Analysis

**Figure 8:**
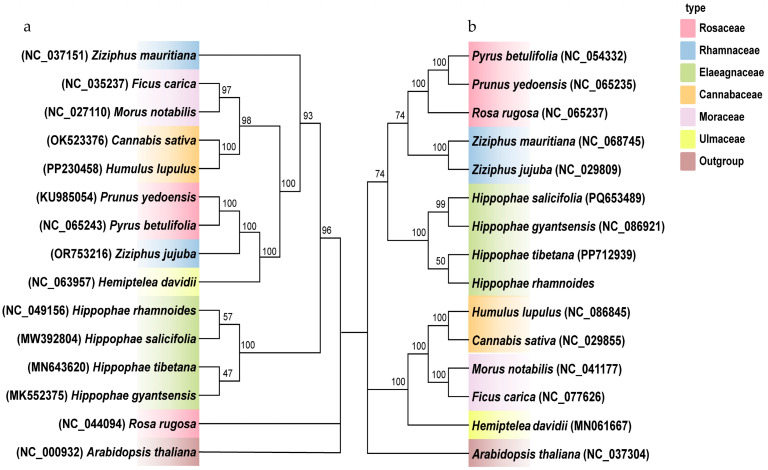
Phylogenetic trees constructed from CDSs from genomes of (**a**) chloroplasts; (**b**) mitochondria. Left and right parts of the figure correspond to (**a**,**b**).

An analysis of the topological structures of the phylogenetic trees from chloroplast and mitochondrial genomes revealed notable consistency in clustering relationships at both the family and genus levels. In the chloroplast phylogenetic tree (Figure 8), *Hippophae* species, including *H. rhamnoides* ssp. *sinensis* and *H. salicifolia*, formed a distinct clade alongside Rosaceae species such as *Prunus yedoensis* and *Pyrus betulifolia*. Similarly, in the mitochondrial phylogenetic tree, *Hippophae* species formed a close branch with Moraceae plants, including *Cannabis sativa* and *Humulus lupulus*. This consistency indicates that chloroplast and mitochondrial genomes synergistically reflect phylogenetic relationships, likely due to shared evolutionary pressures during genetic material transmission.

However, significant differences in support rates at key nodes are evident between the two phylogenetic trees. In the mitochondrial phylogenetic tree (Figure 8), *H. rhamnoides* ssp. *sinensis* shows a close relationship with *Hippophae tibetana*, with a support rate of 50%. In contrast, the chloroplast phylogenetic tree reveals *H. rhamnoides* ssp. *sinensis* being tightly clustered with *Hippophae salicifolia*, with a support rate of 57%. This aligns with findings from the mitochondrial genome study of *Hippophae salicifolia* [28]. In the mitochondrial genome phylogenetic tree, two *Ziziphus* species clustered together, whereas they were distinctly separated in the chloroplast genome phylogenetic tree. Similarly, *Rosa rugosa* (Rosaceae) clustered with *Prunus yedoensis* and *Pyrus betulifolia* in the mitochondrial genome phylogenetic tree but was isolated in the chloroplast genome phylogenetic tree. This discrepancy may arise from distinct genetic characteristics, such as the mitochondrial genome’s maternal inheritance, leading to more specific variations in certain taxa.

### 2.7. Synteny Analysis

**Figure 9:**
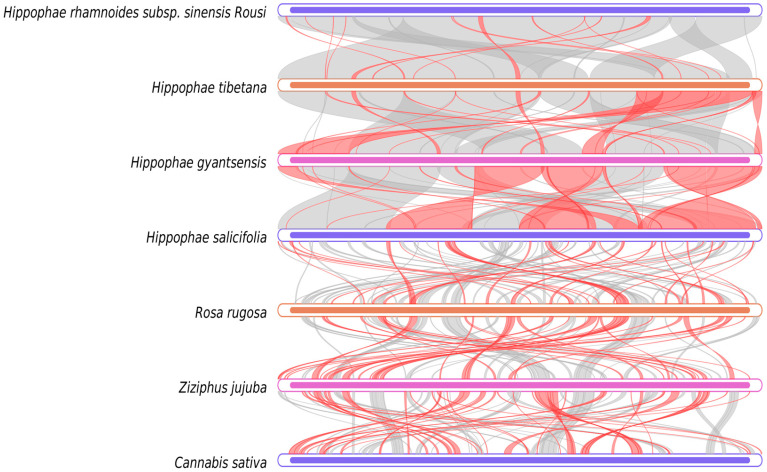
Multiple synteny plot comparing the *H. rhamnoides* ssp. *sinensis* genome with those of closely related species. The boxes in each row represent a genome, and the connecting lines in the middle indicate regions of homology. The red arcs indicate inverted regions, while the gray arcs indicate regions of higher similarity.

The collinearity analysis (Figure 9) showed that the mitochondrial genome sequences of *H. rhamnoides* ssp. *sinensis*, when compared with congeneric species (*Hippophae thibetana*, *H. gyantsensis*, and *H. salicifolia*), were dominated by extensive gray regions with minimal red inversion zones. Notably, the gray region coverage with *H. salicifolia* reached 99.98%, while the coverage with *H. thibetana* and *H. gyantsensis* exceeded 99%, indicating highly conserved genome structures and minimal evolutionary divergence within *Hippophae*. In contrast, comparisons with species from other families (e.g., *Rosa rugosa*, *Ziziphus jujuba*, and *Cannabis sativa*) showed significantly reduced gray regions and increased red inversion zones. For example, the gray region between *H. salicifolia* and *Rosa rugosa* was only 27.85%, while the red inversion zones between *Ziziphus jujuba* and *Cannabis sativa* accounted for 38–44%, indicating extensive genomic rearrangements between different genera.

**Figure 10:**
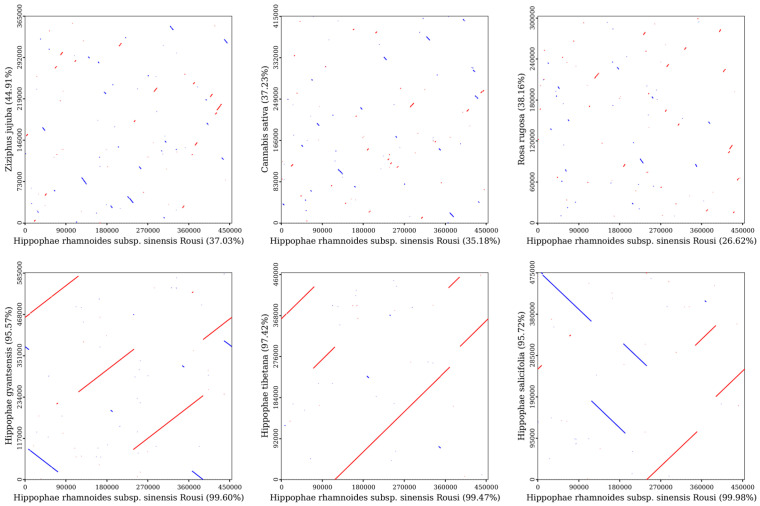
Dot plot analysis of the mitochondrial sequences. In each box, the horizontal axis represents the assembled sequences, while the vertical axis denotes other sequences. The values in parentheses indicate the proportion of homologous sequences relative to the total genome. The red lines within the box illustrate forward alignments, whereas the blue lines depict reverse complementary alignments.

The Dot plot (Figure 10) further confirms dense alignment lines and extensive similar regions among species of the same genus, but sparse alignment lines and scattered similar regions with species from other families or genera. The data show that similar regions between *H. rhamnoides* ssp. *sinensis* and *H. salicifolia* account for 99.98% (454,370 bp) and 95.72% (454,785 bp) of their lengths, while similar regions with *Ziziphus jujuba* are 37.03% (168,288 bp) and 44.91% (164,020 bp), respectively. This confirms highly conserved and closely related mitochondrial genome sequences within *Hippophae*, but significant genomic structural differentiation between species of Rosales families or genera due to evolutionary divergence.

### 2.8. Homologous Fragments of the Mitochondrial and Chloroplast Genomes of H. rhamnoides ssp. sinensis

**Figure 11:**
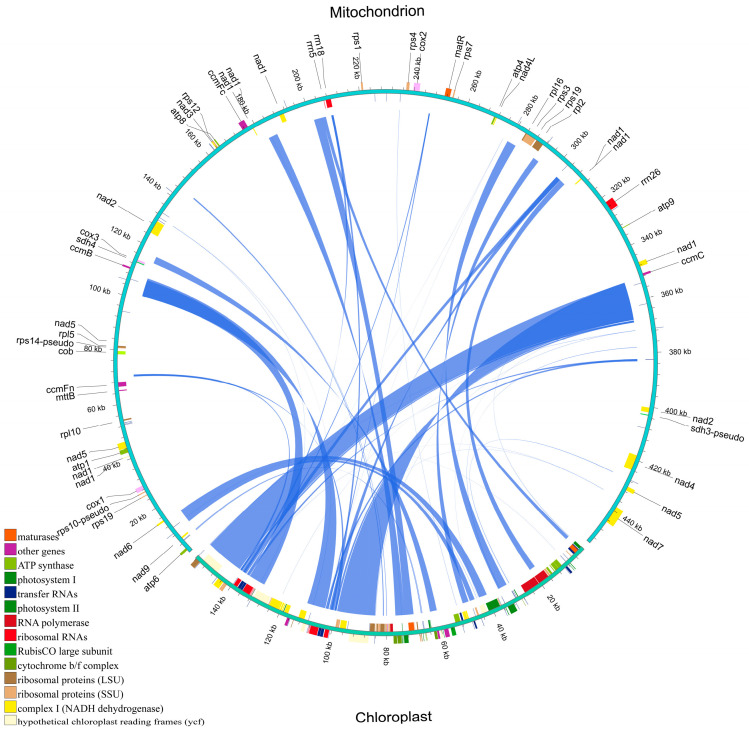
Homologous fragments between chloroplast and mitochondrial gene sequences in *H. rhamnoides* ssp. *sinensis*. Chloroplast and mitochondrial sequences are indicated, with homologous genes from the same complexes being colored identically. Connecting lines in the middle represent homologous sequences.

Our analysis of the mitochondrial and chloroplast genomes of *H. rhamnoides* ssp. *sinensis* revealed a substantial number of homologous fragments. These homologous fragments span 77,741 bp in the chloroplast genome (49.72% of 156,355 bp) and 53,872 bp in the mitochondrial genome (11.85% of 454,444 bp) (Figure 11 and Supplementary Materials Table S7). Compared to most plants, the higher proportion of homologous sequences in the chloroplast genome may indicate more active genetic exchange between the two organelles.

The analysis identified numerous genes with homologous sequences. tRNA genes such as *trnL-CAA* and *trnI-CAU* exhibit highly similar homologous sequences between the two genomes, with some being entirely located within homologous fragments. For example, *trnL-CAA* exhibits 98.247% similarity between the chloroplast and mitochondrial genomes, with an alignment length of 14,950 bp (Supplementary Materials Table S8). Homologous sequences are also present in protein-coding genes, including photosynthesis-related genes (e.g., *psbB* and *psbD*) and NADH dehydrogenase-related genes (e.g., *ndhB* and *ndhF*). The *psbB* gene is partially located within a homologous region. A comparative analysis indicated that the *psbB* gene may have undergone sequence sharing or transfer events during evolution, as indicated by the similarity and alignment length. Partial protein sequences exhibit homology between the chloroplast and mitochondrial genomes. For example, the *cemA* protein exhibits 99.134% similarity in amino acid sequence alignment, with an alignment length of 231 and minimal mismatches or gaps (Supplementary Materials Table S9). This confirms the close functional relationship between the two genomes at the protein level, indicating synergistic roles of these proteins in organelle function.

### 2.9. RNA Editing Events

**Figure 12:**
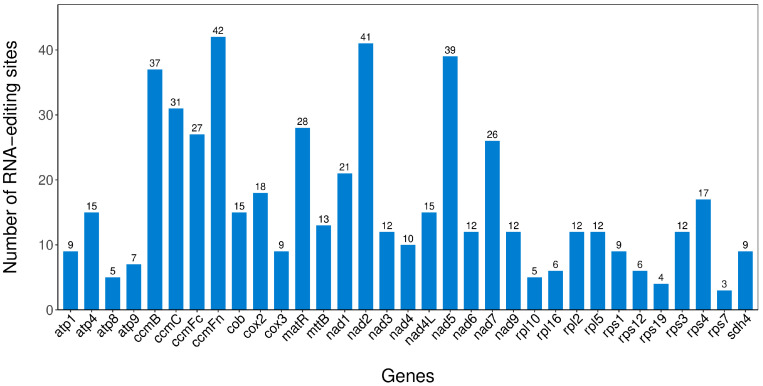
Distribution of RNA editing sites in protein-coding genes of the mitochondrial genome of *H. rhamnoides* ssp. *sinensis*.

Our analysis of the mitochondrial genome of *H. rhamnoides* ssp. *sinensis* revealed 539 RNA editing sites distributed across multiple genes (Supplementary Materials Table S10). The number of RNA editing sites varied significantly among genes (Figure 12), with *ccmFn* exhibiting the highest (42 sites) and *rps7* the lowest (3 sites). This variation indicates distinct regulatory mechanisms for RNA editing, which are potentially linked to the functional importance, expression levels, and evolutionary history of genes.

Types of RNA editing types include hydrophilic-to-hydrophilic, hydrophilic-to-hydrophobic, hydrophilic-to-stop codon, hydrophobic-to-hydrophilic, and hydrophobic-to-hydrophobic conversions (Supplementary Materials Table S11). Hydrophilic-to-hydrophobic conversions are the most frequent (246 occurrences, 45.64% of all events), followed by hydrophobic-to-hydrophobic conversions (190 occurrences, 35.25%). Hydrophilic-to-hydrophilic conversions occur 56 times (10.39%), while hydrophobic-to-hydrophilic conversions occur 43 times (7.98%). Hydrophilic-to-stop codon conversions are the least frequent (four occurrences, 0.74%). RNA editing in the mitochondrial genome of *H. rhamnoides* ssp. *sinensis* exhibits remarkable diversity in the number and types of editing sites among genes. These editing events significantly impact amino acid sequences and may play a crucial role in mitochondrial function, environmental adaptation, and species evolution.

## 3. Discussion

### 3.1. Conservation of Mitochondrial Genome Structure and Function

The mitochondrial genome of *H. rhamnoides* ssp. *sinensis* contains 37 protein-coding genes (PCGs), 30 tRNA genes, 3 rRNA genes, and 3 pseudogenes, showing high similarity in gene composition to other seabuckthorn species, including *Hippophae thibetana* and *Hippophae salicifolia* [28,29]. However, *H. rhamnoides* ssp. *sinensis* lacks the *rps2* gene that is present in other seabuckthorn species, suggesting lineage-specific gene degeneration. A similar phenomenon occurs in *Hippophae thibetana*, which harbors multiple pseudogenes and variations in gene copy numbers in its mitochondrial genome [29]. This gene loss or functional degeneration likely stems from environmental adaptations during the prolonged evolution of seabuckthorn species.

Throughout the evolution of plant mitochondrial genomes, their gene composition and function demonstrate a balance between conservation and specificity. Most plant mitochondrial genomes retain genes encoding core respiratory chain proteins (e.g., *cox1*, *nad5*), which are essential for energy metabolism [31,32]. Notably, the multicopy tRNA genes in the mitochondrial genome of *H. rhamnoides* ssp. *sinensis* (e.g., four copies of *trnM-CAT*) align with *Hippophae thibetana* [29], contrasting sharply with Rosaceae plants [11]. This discrepancy likely reflects the energy metabolism demands of *Hippophae* species in high-altitude environments, where multicopy tRNA genes enhance the efficiency of mitochondrial protein synthesis supporting respiratory chain function under extreme conditions such as low temperature and hypoxia [33,34]. In Rosaceae plants, dynamic changes in the mitochondrial gene composition are particularly significant. For example, the mitochondrial genome of *Photinia serratifolia* lacks *rps2* and *rps11*, whose functions may be compensated for by means of nuclear gene transfer [32]. A comparative analysis of 38 Rosaceae mitochondrial genomes by Nanjing Agricultural University revealed widespread gene loss and rearrangement events. For instance, Rosoideae species have lost *rpl5*, *rpl16*, and *sdh3*, reflecting genomic streamlining that is closely linked to the rapid evolutionary radiation of Rosaceae plants [11].

### 3.2. Codon Usage Bias and Adaptation

The mitochondrial genome of *H. rhamnoides* ssp. *sinensis* displays a significant non-random codon usage bias, aligning with the general characteristics of plant mitochondrial systems [35]. For example, the high frequencies of the alanine codon GCU (RSCU = 1.5811) and the isoleucine codon AUU (RSCU = 1.3269) likely reflect translational selection, where natural selection favors codons that are recognized by high-abundance tRNAs to enhance the translation efficiency. This theory was validated in classic studies, including that by Ikemura (1981), who established the correlation between codon usage and tRNA abundance [36]. Sharp and Li (1987) introduced the “Codon Adaptation Index” (CAI) to quantify translational selection strength [37]. In plant mitochondria, codon usage patterns in terrestrial plant mitochondrial genomes are relatively stable, likely driven by natural selection, which further supports translational selection [38]. The phenylalanine codon UUU (RSCU = 1.1140) shows a pronounced preference. An analysis of *Ramalina sinensis* and *Ramalina intermedia’s* mitochondrial genomes revealed that UUU (Phe) is the most frequent codon in *Ramalina intermedia*, with both genomes exhibiting similar characteristics [39]. This preference is closely linked to the AT-rich genomic characteristics of plant mitochondria, as many mitochondrial genomes exhibit an AT bias. For example, the slight AT preference in the mitochondrial genome of *Astragalus membranaceus* var. *mongholicus* aligns with angiosperm characteristics [40], while the base composition of *Ramalina sinensis* (A: 33.8%, T: 35.5%) also demonstrates an AT bias [39]. However, *Hippophae tibetana* and *Hippophae salicifolia* demonstrate a distinct AU codon preference, contrasting with that of *H. rhamnoides* ssp. *sinensis* [28,29].

Codon usage bias arises from the interplay of multiple evolutionary mechanisms. Studies suggest that the unique base composition bias of the mitochondrial genome, acting as a significant mutational pressure, directly influences codon selection. Extensive studies on mitochondrial genomes across diverse biological groups support this view [41,42]. In contrast, natural selection drives highly expressed genes to preferentially use “optimal codons” corresponding to abundant tRNAs, thereby enhancing the translation efficiency [36]. Additionally, the unique genetic mode of plant mitochondria may promote neutral evolution via genetic drift, further influencing codon usage patterns [43,44]. This complex codon bias potentially regulates the efficiency of the mitochondrial gene expression efficiency, thus impacting cellular energy metabolism [38], and may reflect molecular mechanisms of species adaptation to environmental changes over evolutionary time [45]. These findings provide critical insights into the evolutionary dynamics and functional optimization of plant mitochondrial genomes.

### 3.3. The Role of Repetitive Sequences in the Structure and Evolution of the Genome

The plant mitochondrial genome exhibits a diverse array of repetitive sequences, including simple sequence repeats (SSRs), tandem repeats, and interspersed repeats. In the mitochondrial genome of *H. rhamnoides* ssp. *sinensis*, 449 repetitive sequences were identified, predominantly interspersed repeats (252), followed by simple repeats (179) and tandem repeats (18). The frequent distribution of these repetitive sequences is closely linked to genomic structural rearrangements, as evidenced by the mitochondrial genome of cucumber (1.685 Mb), which contains 13% interspersed repetitive sequences. Through homologous recombination, these sequences form three independent circular conformations (a 1556 kb main chromosome and smaller chromosomes of 84 kb and 45 kb), directly illustrating the regulatory role of repetitive sequences in genome plasticity [46]. Notably, these repetitive sequences vary significantly in type, quantity, and distribution among plant species. An analysis of the mitochondrial genome of *Taraxacum mongolicum* identified 22 SSRs, 17 tandem repeats, and a substantial number of dispersed repeats. The distribution patterns of these repeat sequences are closely tied to the species’ adaptive strategies for occupying diverse ecological niches [12]. Repetitive sequences are crucial in plant mitochondrial genomes, mediating recombination and genomic variation, influencing gene expression and genome stability, and significantly contributing to plants’ evolution and environmental adaptation.

Repetitive sequences contribute to genomic structural diversity. A recent study pioneered targeted base editing in plant mitochondrial genomes using the TALEN-DdCBE fusion system. This technology introduced heritable point mutations in the mitochondrial *atp1* and *rrn26* genes, indirectly inducing genomic rearrangements that were mediated by repetitive sequences, including large fragment deletions and duplications, thus highlighting the dynamic instability of the mitochondrial structure [47]. Additionally, research by Que Youxiong and You Chuihuai at Fujian Agriculture and Forestry University showed that the mitochondrial genome of *Gelsemium elegans* consists of two circular chromosomes totaling 406,009 bp (chromosome 1: 261,758 bp; chromosome 2: 144,251 bp) [48]. The study identified 145 repeat sequence pairs, with 4 mediating homologous recombination, yielding one major and five minor conformations. These conformations were confirmed via PCR amplification and Sanger sequencing [48]. These findings demonstrate that repetitive sequences in plant mitochondrial genomes strongly mediate recombination, driving genomic structural changes. This likely serves as a key genetic basis for plants to adapt to diverse environments, enhancing their survival and reproduction in changing conditions.

### 3.4. Ka/Ks Ratio and Nucleotide Diversity Analysis

Our Ka/Ks analysis revealed that the mitochondrial genome of *H. rhamnoides* ssp. *sinensis* has undergone complex selective pressures. Most genes exhibited Ka/Ks values below 1 (0.21–0.72), indicating that purifying selection predominantly stabilizes mitochondrial functional genes. This aligns with the conservative evolutionary pattern of plant mitochondria reported by Palmer et al. (2000) [49]. Notably, respiratory chain genes (*cox1*, *cox2*, *cox3*) showed exceptionally low Ka/Ks values (0.068–0.416), reflecting stringent functional constraints on these essential metabolic genes [5]. Conversely, genes such as *atp4* and *nad4* exhibited outlier Ka/Ks values exceeding 1 (up to 4.35), suggesting adaptive evolution. This resembles the positive selection cases in plant mitochondrial genes reported by Cho et al. (2004) [50], potentially indicating species-specific adaptations. Notably, the *atp4* gene shows highly variable selective pressures (0–4.35) across species, suggesting dynamic evolutionary characteristics in its function [51].

The nucleotide diversity (pi) analysis highlighted significant evolutionary constraints across genes, with pi values ranging from the highly conserved *rrn5* gene (0.00290) to the highly variable *sdh4* gene (0.05291). This variation pattern reflects typical characteristics of plant mitochondrial genomes [44]. The high pi value and 61 variable sites in the *sdh4* gene indicate weak purifying selection, reflecting functional plasticity for environmental adaptation [52]. Conversely, the extreme conservation of the *rrn5* gene underscores the core functional constraints of ribosomal RNA in protein synthesis [44]. The respiratory chain genes (*cox1-3*) showed moderately low pi values (0.01575–0.01637), which is consistent with their critical roles in oxidative phosphorylation [5]. The high conservation of these genes suggests that their encoded proteins’ structure and function are essential for the integrity of the mitochondrial electron transport chain [49]. These findings offer critical insights into mitochondrial genes’ functional differentiation and evolutionary mechanisms.

### 3.5. Characterization and Function of RNA Editing

The detection of 539 RNA editing sites in the mitochondrial genome of *H. rhamnoides* ssp. *sinensis*, along with diverse editing types, highlights the critical role of RNA editing in regulating mitochondrial gene expression in plants. This study reveals significant variation in RNA editing sites across genes, with *ccmFn* showing the highest number (42) and *rps19* the fewest (4). This variation may be linked to the functional importance, expression levels, and evolutionary selection pressures of these genes. Studies suggest that genes that are involved in mitochondrial complex assembly, such as *ccmFn*, often require precise post-transcriptional regulation for proper protein folding and functional maintenance. Conversely, the lower editing frequency in ribosomal protein genes, such as *rps19*, may reflect their higher conservation level [53,54].

Among RNA editing types, hydrophilic-to-hydrophobic conversions are the most common (45.64%), followed by hydrophobic-to-hydrophobic conversions (35.25%). This trend likely reflects the essential role of RNA editing in maintaining the structure and function of proteins. Hydrophilic-to-hydrophobic conversions can profoundly impact protein folding and subcellular localization, thus influencing biological functions [55]. Additionally, hydrophilic-to-stop codon editing events are the rarest, accounting for only 0.74%. However, these events may play a critical role in precise gene expression regulation, such as by preventing premature translation termination to ensure complete protein synthesis [56].

The diversity of RNA editing in the mitochondria of *H. rhamnoides* ssp. *sinensis* may contribute to its environmental adaptability. Studies suggest that RNA editing plays a crucial role in plants’ responses to environmental stresses, such as drought and low temperatures, by potentially optimizing energy metabolism through mitochondrial protein modifications [57]. Future research should explore the dynamic changes in RNA editing in *H. rhamnoides* ssp. *sinensis* in response to environmental factors, aiming to elucidate the mechanisms underlying species adaptation and evolution.

### 3.6. Phylogenetic Analysis and Evolutionary Relationship

Phylogenetic analysis using mitochondrial genomes is a robust approach for elucidating interspecific relationships in plants and has proven valuable in numerous botanical studies. The formation and differentiation of *Hippophae* species likely involved complex hybridization or introgression events. For example, studies on interspecific gene flow in *Tibetan Plateau* plants suggest that species such as *Hippophae gyantsensis* originated through homoploid hybridization, with the gene flow significantly contributing to *Hippophae* genus differentiation [58,59]. It is hypothesized that *H. rhamnoides* ssp. *sinensis* and *Hippophae tibetana* underwent hybridization during recent speciation events, introducing mitochondrial genome components from *H. rhamnoides* ssp. *sinensis* into *Hippophae tibetana*, which explains their closer clustering in the evolutionary tree.

The chloroplast genome, characterized by maternal inheritance and structural stability, provides a more accurate reflection of the long-term evolutionary genetic relationships between species. This maternal inheritance is particularly evident in bananas (*Musa acuminata*), as studies have shown that the chloroplast genome follows strict maternal inheritance [60]. The chloroplast phylogenetic tree analysis by Jia and Bartish (2018) reveals that *H. tibetana* and *H. rhamnoides* form a distinct clade, while *H. gyantsensis*, *H. salicifolia*, and *H. neurocarpa* constitute a weakly supported clade [61]. These results significantly enhance our understanding of the evolutionary dynamics and biogeographic patterns of the *Hippophae genus* and advance knowledge of phylogenetic relationships within the Rosales order [61]. In seed plants, mitochondrial genes exhibit a relatively low synonymous substitution rate and a slower evolutionary rate compared to chloroplast genes [62]. However, significant differences are observed compared to the phylogenetic tree presented by Zhang et al. (2011), which may result from differences in datasets, data processing methods, and the models and approaches used in tree construction [63]. Studies on the sorghum mitochondrial genome indicate that repetitive sequences and recombination hotspots drive frequent structural variations [64]. Similarly, the mitochondrial genomes of *H. rhamnoides* ssp. *sinensis* and *Hippophae tibetana* may exhibit shorter genetic distances in the phylogenetic tree due to increased similarity in certain regions, likely resulting from recombination or horizontal gene transfer events.

## 4. Materials and Methods

### 4.1. Sample Collection and Sequencing Data

The geographical distribution data of *H. rhamnoides* ssp. *sinensis* were obtained from the Global Biodiversity Information Facility (GBIF; GBIF.org (7 August 2025) GBIF Occurrence Download https://doi.org/10.15468/dl.davr5z (accessed on 7 August 2025)). The plant material for sequencing was *H. rhamnoides* ssp. *sinensis*, a species widely distributed in Qinghai Province, China. The plant material was collected from Bianmagou Road, Datong County, Xining City, Qinghai Province, China (coordinates: 36°36′05.64″ N, 101°48′16.39″ E). The leaf material underwent DNA extraction using the CTAB method, followed by purification with the T3010 kit (QIAGEN, Hilden, Germany). Subsequently, Nanodrop and Qubit assays were conducted, yielding an Nc/Qc ratio of 0.97. The sample quality was deemed satisfactory, meeting the requirements for library construction and sequencing.

The mitochondrial genome was assembled using a combination of second- and third-generation sequencing strategies by Nanjing Jisi Huiyuan Biotechnology Co., Nanjing, China. The second-generation sequencing was conducted following the Illumina standard protocol, which included sample quality testing, library construction, library quality assessment, and sequencing. After passing quality control, the genomic DNA was fragmented through mechanical shearing (ultrasonication). The fragmented DNA was purified, end-repaired, adenylated at the 3′-end, and ligated with sequencing adapters. Fragment size selection was performed using agarose gel electrophoresis, followed by PCR amplification to construct sequencing libraries. The libraries were subjected to quality control, and qualified ones were sequenced on the Illumina NovaSeq X Plus platform (Illumina, San Diego, CA, USA). Paired-end sequencing was employed, utilizing the PE150 (Paired-end 150 bp) method. The raw data were processed using the fastp software (v0.23.4, https://github.com/OpenGene/fastp (accessed on 24 April 2025)) to trim sequencing adapters and primer sequences, remove reads with an average quality score below Q5, and exclude reads containing more than five ambiguous bases (N).

Following quality control, high-quality DNA sequencing libraries were obtained. For third-generation sequencing, genomic DNA was randomly fragmented, and large fragments were enriched and purified using magnetic beads. These fragments were size-selected and recovered. The fragmented DNA was subjected to damage repair, purification, end repair, 3′-end adenylation, and ligation using adapters from the SQK-LSK109 (Nanopore, Oxford, UK) ligation kit. The DNA library was quantitatively assessed. After construction, the DNA library was loaded onto the Flow Cell (R9.4.3) at an appropriate concentration and volume, followed by transfer to the Oxford Nanopore PromethION (Nanopore, Oxford, UK) sequencer for real-time single-molecule sequencing. The third-generation sequencing data were filtered using Filtlong software (v0.2.1, https://github.com/rrwick/Filtlong (accessed on 24 April 2025)) with the following parameters: --min_length 1000 --min_mean_q 7. Basecalling was performed using Guppy (https://community.nanoporetech.com/downloads (accessed on 24 April 2025)) with the run mode set to high accuracy mode (hac). Perl scripts were employed for additional data filtering and statistical analysis of the third-generation sequencing results (https://github.com/rrwick/Filtlong (accessed on 24 April 2025)).

### 4.2. Genome Assembly

Plant mitochondrial genes (CDS and rRNA) are highly conserved. Leveraging this feature, we used minimap2 (v2.1) [65] to align the original third-generation sequencing data with reference gene sequences (plant mitochondrial core genes, https://github.com/xul962464/plant_mt_ref_gene (accessed on 25 April 2025)) and selected sequences that were longer than 50 bp as candidates. From these candidates, sequences containing multiple core genes and demonstrating higher alignment quality (with more complete core genes covered) were selected as seed sequences. Next, minimap2 was used to map the original third-generation sequencing data to the seed sequences, identify sequences with overlaps larger than 1 kb, and iteratively add them to the seed sequences to extract all reads corresponding to the mitochondrial genome. The extracted third-generation data were corrected using Canu (snapshot v1.6 +13) [66]. Subsequently, the second-generation sequencing data were aligned to the corrected sequences using Bowtie2 (v2.3.5.1) [67], followed by hybrid assembly with Unicycler (v0.4.8) (https://github.com/rrwick/Unicycler (accessed on 25 April 2025)) using default parameters to integrate the second-generation and corrected third-generation data. The assembly results were visualized and manually refined using Bandage (v0.8.1) [68]. Given the potential for multiple sub-loops or complex non-circular structures in the mitochondrial genome, the corrected third-generation sequencing data were compared to the Unicycler-assembled contigs using minimap2 to manually determine the orientation and branching structure, yielding the final assembly results.

### 4.3. Genome Annotation and Other Analyses

Mitochondrial annotation was conducted through the following steps: protein-coding genes and rRNAs were compared to published and reference plant mitochondrial sequences using BLAST (version 2.14.0) and manually curated based on closely related species. tRNAs were annotated using tRNAscan-SE (version 2.0) [69] (http://lowelab.ucsc.edu/tRNAscan-SE/ (accessed on 25 April 2025)). Open reading frames (ORFs) were identified using the Open Reading Frame Finder (http://www.ncbi.nlm.nih.gov/gorf/gorf.html (accessed on 25 April 2025)) with a minimum length threshold of 102 bp to exclude redundant sequences and those overlapping with known genes. Sequences that were longer than 300 bp were annotated by searching against the NR database. RNA editing sites were predicted using PmtREP (http://cloud.genepioneer.com:9929/#/tool/alltool/detail/336 (accessed on 25 April 2025)). The results were reviewed and manually refined to generate the final annotation.

### 4.4. Codon Preference Analysis

Due to codon degeneracy, each amino acid is encoded by a minimum of one and a maximum of six codons. Genomic codon usage varies significantly across species and organisms. This disparity in synonymous codon usage is termed Relative Synonymous Codon Usage (RSCU). This preference is thought to arise from the combined effects of natural selection, species-specific mutations, and genetic drift. RSCU is calculated as the ratio of the observed frequency of a specific codon to its expected frequency under the assumption of equal usage of all synonymous codons for that amino acid. Specifically, RSCU is calculated by dividing the number of occurrences of a codon for an amino acid by the total number of codons for that amino acid and then dividing this by the reciprocal of the number of synonymous codons for that amino acid, i.e., RSCU = (observed frequency of codon usage)/(theoretical frequency of codon usage). Unique coding sequences (CDSs) were screened and analyzed using a Perl (version 5.38.0) script (http://cloud.genepioneer.com:9929/#/tool/alltool/detail/214 (accessed on 26 April 2025)).

### 4.5. Repeat Sequence Analysis

Repeated sequences encompass simple sequence repeats (SSRs), tandem repeats, and dispersed repeats. SSRs were identified using MISA software (v1.0) [70] with the following parameters: 1-10, 2-5, 3-4, 4-3, 5-3, and 6-3. Tandem repeats were identified using TRF software (trf409.linux64) [71] with the following parameters: 2 7 7 80 10 50 2000 -f -d -m. Dispersed repeats were identified using BLASTN (v2.10.1) [72] with the following parameters: -word_size 7, evalue 1 × 10^−5^. Finally, redundancy and crosstalk duplicates were removed during post-processing. The results were visualized using Circos v0.69-5 (http://circos.ca/software/download/ (accessed on 26 April 2025)).

### 4.6. Assessment of ka/ks Ratio

A base variation resulting in an amino acid change is termed a nonsynonymous mutation, while one that does not alter the amino acid is referred to as a synonymous mutation. Nonsynonymous mutations are typically influenced by natural selection. The ratio of the nonsynonymous mutation rate (Ka) to the synonymous mutation rate (Ks) reflects the type of selection pressure acting on the mutations. A Ka/Ks ratio greater than 1 suggests positive selection, whereas a ratio below 1 indicates purifying selection. Advanced analytical species were grouped into pairs, and homologous gene pairs were extracted and aligned using the MAFFT v7.427 software (https://mafft.cbrc.jp/alignment/software/ (accessed on 27 April 2025)) [73]. After alignment, Ka and Ks values for each gene pair were calculated using the KaKs_Calculator v2.0 software (https://sourceforge.net/projects/kakscalculator2/ (accessed on 27 April 2025)) [74] with the MLWL method. Finally, the Ka/Ks values for each gene pair were compiled, and a boxplot was constructed.

### 4.7. Nucleotide Diversity (Pi) Analysis

The nucleotide diversity (pi) serves as a measure of variation in nucleic acid sequences across different species. Regions exhibiting higher variability may serve as potential molecular markers for population genetics. Global alignment of homologous gene sequences from various species was conducted using the MAFFT software (v7.427, --auto mode), while DnaSP5 was utilized to calculate the pi value for each gene [75].

### 4.8. Evolutionary Tree Analysis

The maximum likelihood evolutionary tree was constructed based on coding sequences (CDSs). MAFFT (v7.427, --auto mode) was used for multiple sequence alignment. The aligned sequences were concatenated and trimmed using trimAl (v1.4.rev15) [76] with a gap threshold of 0.7. Following trimming, the best-fit model (GTR) was identified using jModelTest 2.1.10 (https://github.com/ddarriba/jmodeltest2 (accessed on 28 April 2025)) [77]. The maximum likelihood tree was then generated using RAxML v8.2.10 (https://cme.h-its.org/exelixis/software.html (accessed on 28 April 2025)) with the GTRGAMMA model and 1000 bootstrap replicates. Additionally, all selected chloroplast genomes were aligned using the MAFFT plug-in in PhyloSuite (http://phylosuite.jushengwu.com/dongzhang0725.github.io/ (accessed on 23 May 2025)). Conserved regions were identified and extracted with Gblocks. The extracted sequences were concatenated. The most suitable substitution model was selected using ModelFinder. The maximum likelihood tree was constructed with IQ-TREE. Finally, the phylogenetic tree was visualized and enhanced by importing the tree file into ChiPlot (https://www.chiplot.online/ (accessed on 23 May 2025)) [78].

### 4.9. Mitochondrial Sequence Covariance Analysis

Pairwise alignments between the assembled and selected species were performed using the blastn software (version 2.10.1 or later) with a word size of 7 and an E-value threshold of 1 × 10^−5^, generating a synteny plot. Genome alignments between other sequences and the assembled sequences were conducted using the nucmer software (version 4.0.0 beta2) [79] with the --maxmatch option, resulting in a Dot plot. In the Dot plot, the *x*-axis represents the assembled sequences, and the y-axis corresponds to the other sequences. Forward alignments are depicted by red lines, while reverse complementary alignments are shown with blue lines within the boxes.

### 4.10. Chloroplast and Mitochondrial Homologous Sequence Analysis

Homologous sequences between chloroplasts and mitochondria were identified using the BLAST software with a similarity threshold of 70% and an E-value of 1 × 10^−5^, while keeping all other parameters at their default settings. The homologous fragments between the chloroplast and mitochondrial genomes were visualized using Circos v0.69-5 for intuitive display. Additionally, the amino acid sequences of the chloroplast genome were aligned against the plant mitochondrial genome using BLAST software, illustrating the transfer of coding sequences from the chloroplast to the mitochondrial genome.

## 5. Conclusions

For the first time, the mitochondrial genome of *H. rhamnoides* ssp. *sinensis* was comprehensively analyzed, and a circular mitochondrial genome spanning 454,444 bp was assembled and annotated. The mitochondrial genome of *H. rhamnoides* ssp. *sinensis* exhibits typical angiosperm characteristics, comprising 37 protein-coding genes, 30 tRNA genes, 3 rRNA genes, and 3 pseudogenes. Our genomic analysis demonstrated a high conservation of energy metabolism-related genes, particularly those involved in oxidative phosphorylation. Compared with other *Hippophae* species, the mitochondrial genome of *H. rhamnoides* ssp. *sinensis* displayed lineage-specific gene loss, including the deletion of the *rps2* gene.

Significant codon usage biases were observed, including a strong preference for GCU-encoded alanine and a high frequency of the UAA stop codon. The repetitive sequence analysis revealed 449 repetitive sequences, predominantly scattered repeats, which may drive genome evolution via recombination mechanisms. The selection pressure analysis indicated that most genes underwent purifying selection, while genes such as *atp4* and *nad4* exhibited positive selection signals, potentially highlighting their role in *H. rhamnoides* ssp. *sinensis* ’s adaptation to high-altitude environments. Our phylogenetic analysis further confirmed that *H. rhamnoides* ssp. *sinensis* is the closest relative to *Hippophae tibetana*, supporting the taxonomic classification within the genus.

Numerous homologous sequences were identified between the chloroplast and mitochondrial genomes, spanning nearly 50% of the chloroplast genome. This indicates active gene transfer events between the two organelles. Our RNA editing analyses predicted 539 editing sites, predominantly involving changes from hydrophilic to hydrophobic amino acids, which potentially regulate mitochondrial functions through protein property alterations. These findings address gaps in the research on sea buckthorn’s mitochondrial genome and offer novel insights into the evolutionary mechanisms and environmental adaptation of plant mitochondrial genomes. They establish a critical foundation for subsequent genetic improvement and functional genomics studies of *H. rhamnoides* ssp. *sinensis* and provide a valuable case study for plant organelle genome evolution research.

## Data Availability

The original sequencing data have been submitted to the NCBI database and received GenBank accession numbers PV819143. The data used in this study are already entirely in the public domain (https://www.ncbi.nlm.nih.gov).

## Supplementary Materials

The following supporting information can be downloaded at https://www.mdpi.com/article/10.3390/plants14162547/s1: Figure S1: Base content distribution chart. Figure S2: Error rate distribution chart. Figure S3: Quality value distribution chart. Figure S4: Reads’ length distribution chart. Figure S5: Type and proportion of simple sequence repeats (SSRs). Figure S6: Different types of nucleotide motifs. Figure S7: The length distribution of dispersed repeat. Table S1: Statistical table of second-generation and third-generation sequencing data for *H. rhamnoides* ssp. *sinensis*. Table S2: Functional classifications and physical locations of genes in the mitochondrial genome of *H. rhamnoides* ssp. *sinensis*. Table S3: Statistical analysis of gene functions in *H. rhamnoides* ssp. *sinensis*. Table S4: Codon usage of amino acid pairs in the mitochondrial genomes of *H. rhamnoides* ssp. *sinensis*. Table S5: Distribution of tandem repeats in the mitochondrial genome of *H. rhamnoides* ssp. *sinensis*. Table S6: The Ka/Ks values of each gene pair between *H. rhamnoides* ssp. *sinensis* and the other six species. Table S7: Statistical analysis of homologous sequences in chloroplasts and mitochondria of *H. rhamnoides* ssp. *sinensis*. Table S8: Homologous fragments between mitochondria and chloroplasts in *H. rhamnoides* ssp. *sinensis*. Table S9: Comparative analysis of amino acid sequences between chloroplast and mitochondrial genomes of *H. rhamnoides* ssp. *sinensis*. Table S10: Prediction of RNA editing sites in the mitochondrial genome of *H. rhamnoides* ssp. *sinensis*. Table S11: Statistical table of amino acid hydrophobicity changes induced by RNA editing.
