# Supplementary material for: Characterization of the Mitochondrial Genome of Hippophae rhamnoides subsp. sinensis Rousi Based on High-Throughput Sequencing and Elucidation of Its Evolutionary Mechanisms
Source: Plants (Basel). 2025 Aug 15;14(16):2547. doi: 10.3390/plants14162547 (PMC12389370; doi:10.3390/plants14162547)
Supplement: Supplementary file 1 [file plants-14-02547-s001.zip › Supplementary Material Figures.pdf]

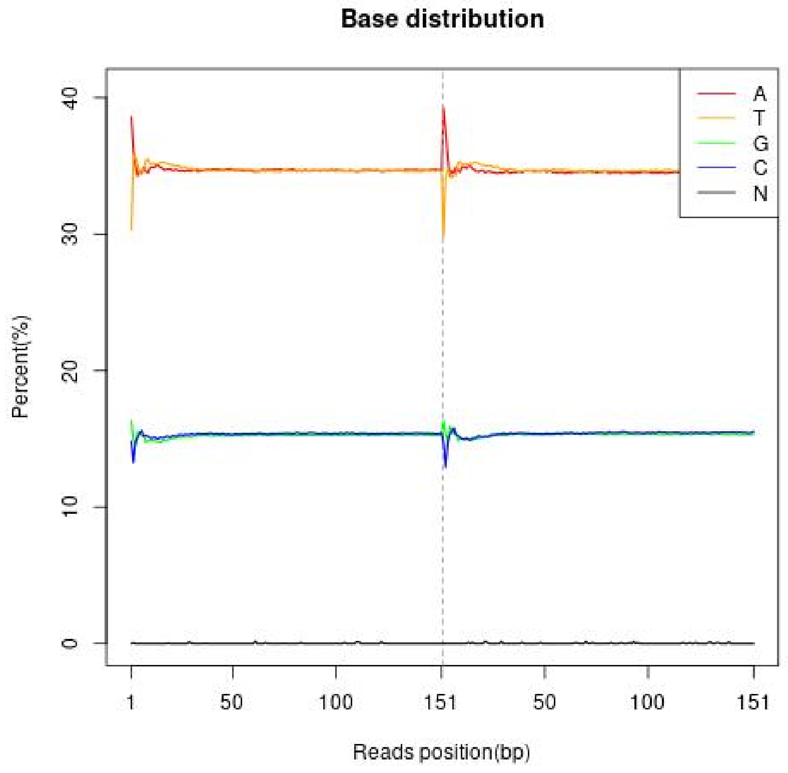

Supplementary Figures S1: Base content distribution chart

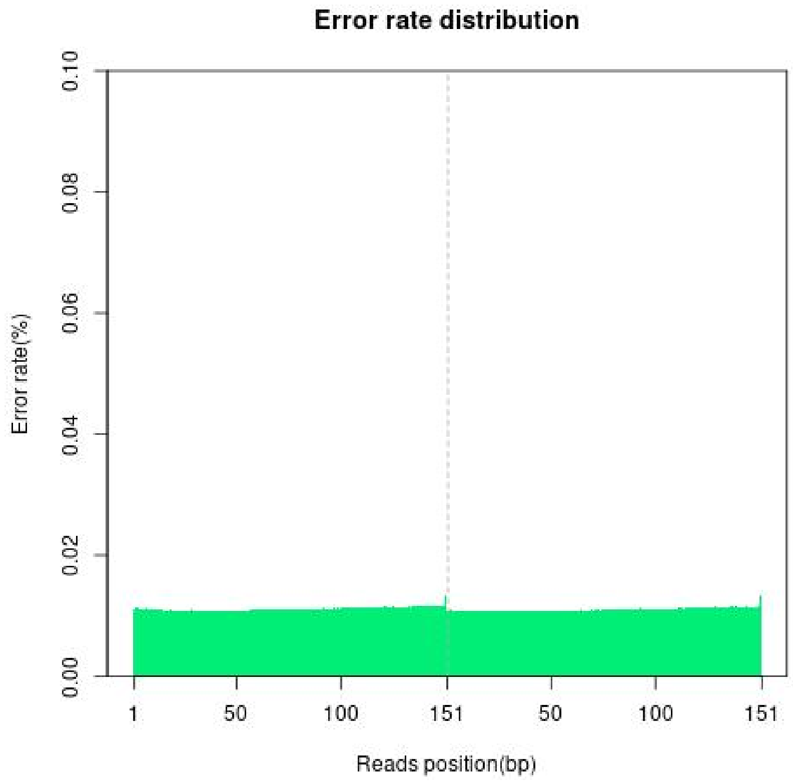

Supplementary Figures S2: Error rate distribution chart

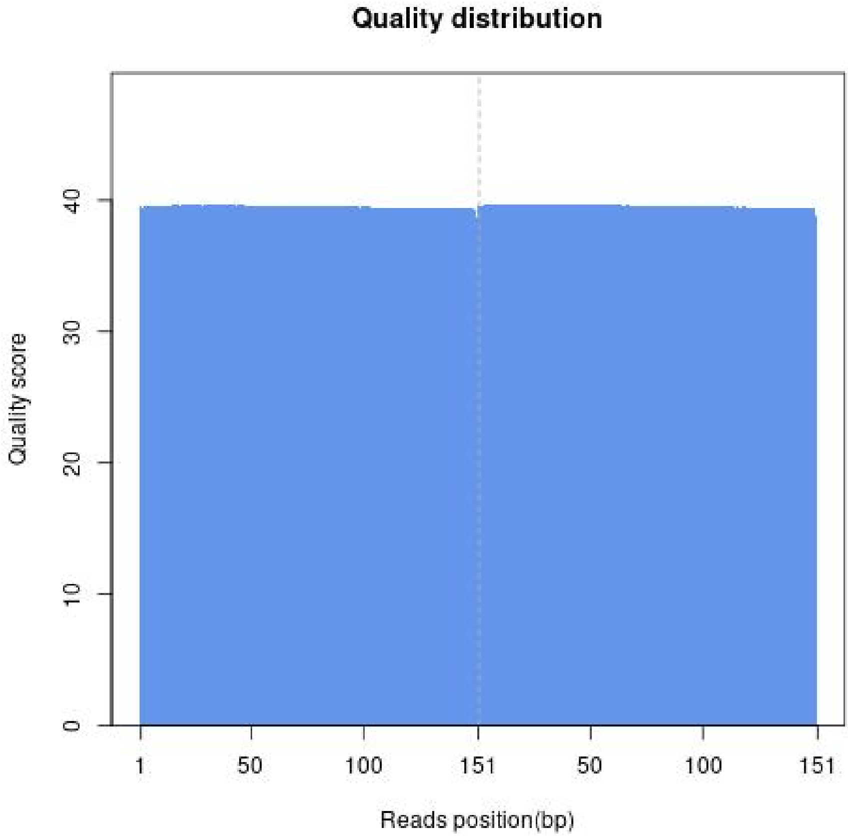

Supplementary Figures S3: Quality value distribution chart

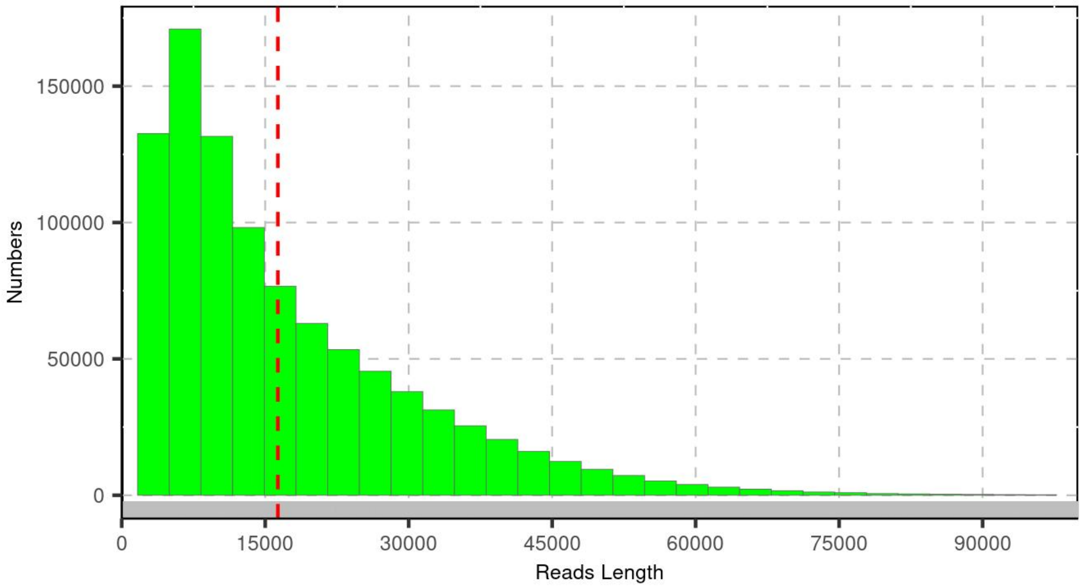

Supplementary Figures S4: Reads length distribution chart

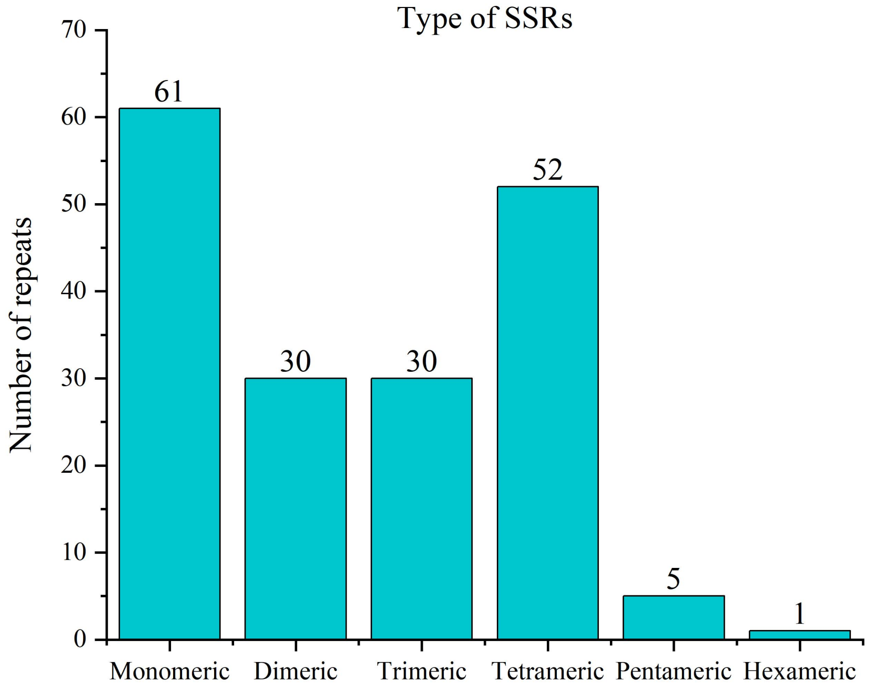

Supplementary Figures S5: Type and proportion of simple sequence repeats (SSRs)

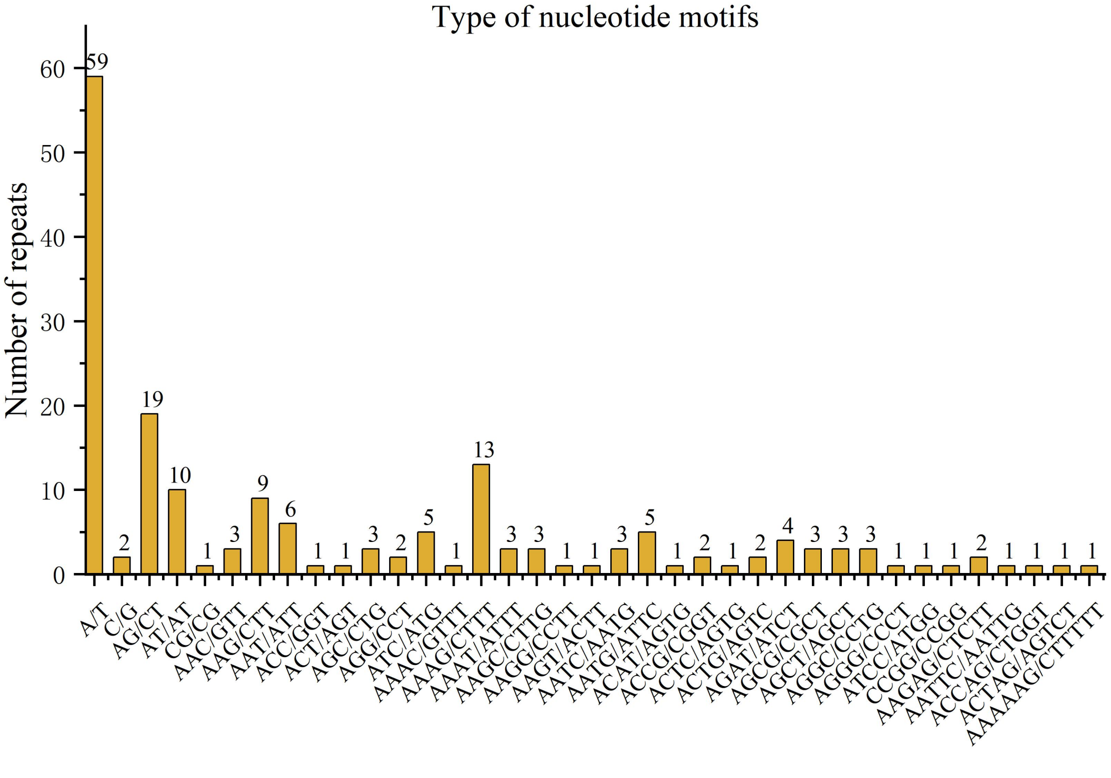

Supplementary Figures S6: Different types of nucleotide motifs

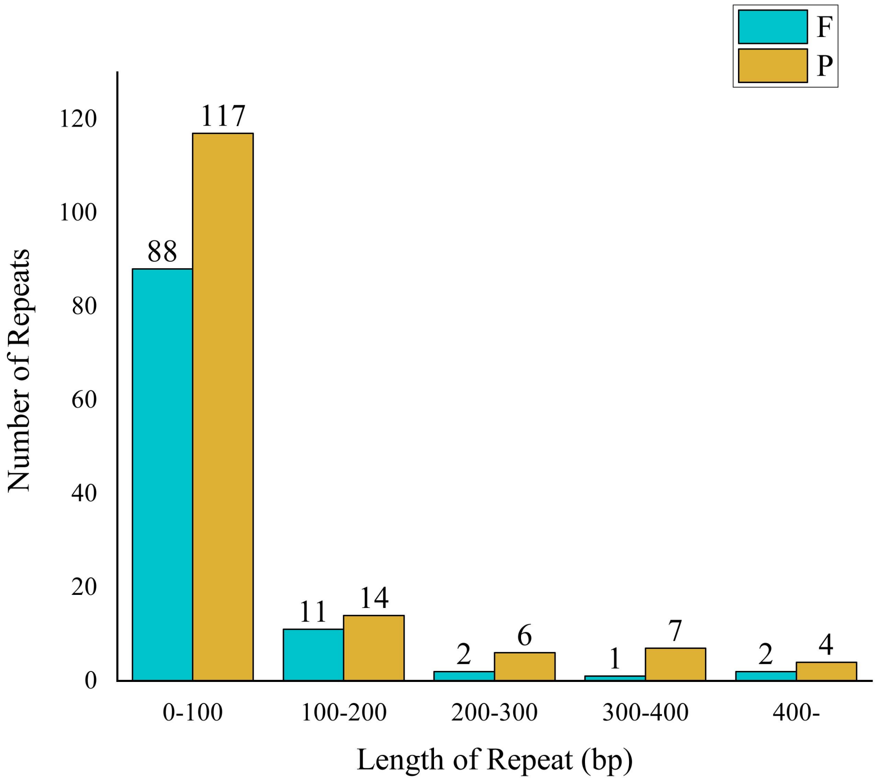

Supplementary Figures S7: The length distribution of dispersed repeat. The blue and yellow lines represent forward (F) and palindromic (P)
